# Fracture distribution in cross-country skiing accidents: an observational study from the Swedish Fracture Register

**DOI:** 10.1007/s00590-026-04695-0

**Published:** 2026-03-06

**Authors:** Björn Hernefalk, Anders Brüggemann, Olof Wolf

**Affiliations:** Department of Surgical Sciences, Section of Orthopaedics, Uppsala University, Uppsala, Sweden

**Keywords:** Fracture, Orthopaedic trauma, Fracture distribution, Cross-country skiing, Swedish fracture register, Skiing

## Abstract

**Background and purpose:**

Cross-country skiing (CCS) is a popular winter activity in Sweden, but little is known about what type of orthopaedic injuries are sustained following accidents in the general population. The purpose of this study was to describe the fracture distribution in accidents during cross-country skiing.

**Methods:**

In this registry-based observational study, all patients who sustained one or more fractures in CCS accidents registered in the Swedish Fracture Register (SFR) with injury date between January 1, 2015 and June 30, 2022 were included. Fractures were classified using the AO Foundation/Orthopaedic Trauma Association (AO/OTA) fracture classification system. The fracture distribution was described and age and sex differences examined. The proportion of surgically treated fractures per fractured location was assessed.

**Results:**

During the study period, 1820 fractures in 1766 patients were registered in the SFR. The median age of patients was 56 years, and 63% of fractures occurred in females. Wrist fractures were most common (22%), followed by hand fractures (19%). Upper extremity fractures accounted for 66% of all fractures. Operative treatment was performed on 560 (31%) fractures, but most fractures were treated non-operatively in both adults (n=1070, 64%) and children (n=113, 79%).

**Interpretation:**

Fractures following CCS injures are more common in females than in males. Most fractures affect the upper extremity and are treated nonoperatively.

## Introduction

### Background

Cross–country skiing (CCS) is a popular winter activity in Sweden, appealing to a broad spectrum of individuals, ranging from professional athletes to those who ski solely for recreational purposes. For the latter, CCS provides a good way to get exercise during the winter months.

There are two main techniques in CCS, classical and skate. First mentioned in the 1930s, the popularity of the skating technique has recently increased [1]. In the classical style, skis are kept parallel to each other, using diagonal strides to move forward in a gliding motion, while the skating style consists of an ice-skating motion to alternating sides. In both techniques, poles are used. Since the introduction of the skating technique the speed of the sport has increased [1], but despite this, injury rates have been considered low.

Examining the literature, reports are not conclusive regarding the most common injuries. Some have suggested ankle ligament sprains and fractures, muscle ruptures, and knee ligament sprains [2]. Others report sprains of the medial collateral ligament of the knee, and ulnar collateral ligament sprains of the thumb (skier’s thumb) [3].

Most reports suggest that CCS is a relatively safe sport that offers many health benefits [4–8]. Even though considered safe, injuries do occur, and have been reported to 0.1 to 0.8 injuries per 1,000 skier days [9]. In recreational skiers, an injury rate of 0.51 per 1000 skier days has been described, with a higher rate in women (0.65 per 1000 days for women and 0.40 per 1000 days for men) [10].

During the Olympic tournament in Beijing 2022, the lowest number of injuries among all disciplines was reported in disciplines involving CCS [11]. Of the CCS-related injuries, strains were the most common injuries, followed by fractures. Ankle and lower back injuries were the most common localizations. To what extent this investigation on professional athletes is valid for the general population of cross-country skiers is not known. There are also reports describing the fracture distribution obtained following accidents when slipping on ice or snow [12], but the transferability of those findings to accidents while cross-country skiing is not known.

In summary, the current literature regarding injuries in CCS is scarce. There is a lack of studies reporting population-based fracture distribution specific to cross-country skiing. This limits information regarding what burden of fractures these injuries amount to in the general population and how fractures are distributed and treated.

### Objectives

This study aimed to address the following research questions:

1. What types of fractures are common following CCS accidents in the general population, i.e. what is the distribution of fractures following CCS accidents?
2. Does age and sex impact the risk of sustaining fractures in CCS accidents?
3. What proportion of CCS injuries require surgical treatment?

## Patients and methods

### Study design and setting

This observational study was based on data from the Swedish Fracture Register (SFR). The SFR collects data regarding injury date and mechanism, fracture classification and treatment (operative and non-operative) which is registered by the treating orthopaedic surgeon [13]. Fractures are classified according to the AO/OTA classification (2007 version) [14]. The accuracy of the SFR has been investigated and deemed accurate in several studies [15–17]. The coverage of the SFR has gradually increased to full national coverage in 2021 (54 of 54 orthopaedic departments actively register fractures).

A comparison with the National Patient Register (NPR) in 2022 demonstrated a completeness of the SFR of approximately 60% for all fractures and 81% for femoral fractures. These figures are conservative as the NPR overestimates the number of fractures, mostly due to miscoding old fractures as new at follow-up visits [18].

### Patients and outcome variables

All patients with fractures sustained in CCS accidents between January 1st 2015 and June 30th 2022 and registered in the SFR were included in the study. Baseline data included demographics (age and sex), injury date, fracture localization and classification, presence of open fracture and primary treatment (operative or non-operative). All these were treated as one entity per fracture, not per person. Patients younger than 18 years were classified as children/adolescents.

The fracture classification was used to group fractures into the following anatomical regions: clavicle, scapula, humerus, forearm (including the distal radius), hand, spine, pelvis, femur, patella, tibia, ankle and foot. Sub-analyses included segmental localization of fractures in long bones: proximal, diaphyseal and distal parts. The primary outcome investigated was the distribution of fractures, expressed as percentage for the respective body part in relation to all fractures. Furthermore, the proportion of operatively treated fractures was investigated.

### Ethics, funding, data presentation and potential conflicts of interest

Ethical approval was obtained from the Swedish Ethical Review Authority (Dnr 2022-04355-01). No individual patient consent was needed according to the Swedish Patient Law as National Quality Registers work according to an opt-out principle. Due to legislation with respect to register data and restrictions stipulated in the ethical permission, the dataset analysed in this study is not freely available. Data can be obtained from the Center of Registers, Västra Götaland if ethical approval is granted. The STROBE recommendations for reporting observational studies were applied [19].

The authors declare no competing interests related to this body of work.

### Statistics

Baseline demographic data are presented as the number of fractures, medians with inter-quartile ranges (IQR) and proportions (%). All statistical analyses were performed using R version 4.2.2 [20].

## Results

During the study period, a total of 1820 fractures in 1766 patients were reported. The median age was 56 years and fractures were more common in females (*n* = 1151, 63%). There were 143 fractures in children/adolescents, accounting for 7.8% of fractures.

Most fractures were closed (*n* = 1816, 99.8%) (Table 1). Data regarding treatment (operative or non-operative) was available for 1743 (95.8%) of fractures.

**Table 1:** Characteristics of 1766 patients with 1820 fractures sustained by cross-country skiing

Patients	1766
Fractures	1820
Median age (IQR)	56 (23)
Sex	
Fractures in males	669 (37%)
Fractures in females	1151 (63%)
Adult fractures	1677
Fractures in children/adolescents. (< 18 years)	143
Open fractures	4 (0.2%)

## Fracture distribution

### Overall fracture distribution

**Fig. 1:**
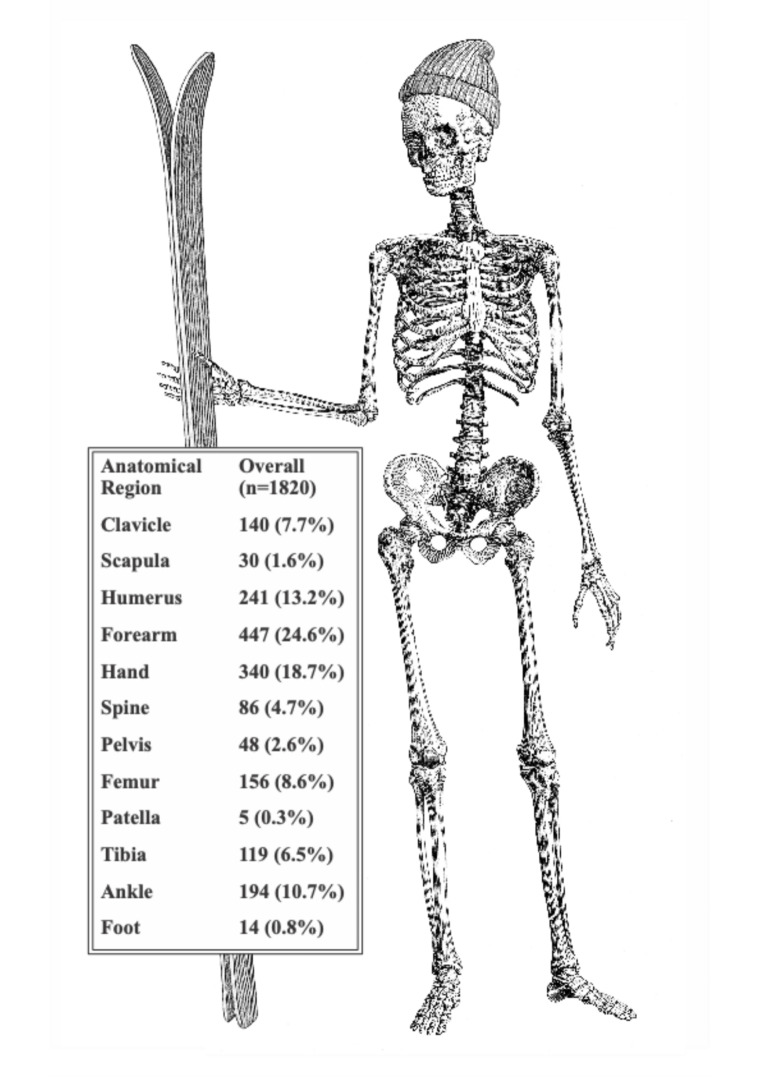
Fracture distribution in cross country skiing

Wrist fractures were most common (*n* = 399, 21.9%), followed by fractures of the hand (*n* = 340, 18.7%). Fractures of the upper extremity comprised 65.8% of all fractures, whereas the lower extremities accounted for 29.4% of injuries. (Fig. 1, Table 2)

**Table 2:** Fracture distribution in injuries sustained by cross-country skiing for children, adults and overall

Anatomical Region	Adults (*n* = 1677)	Children (*n* = 143)	Overall (*n* = 1820)
Clavicle	132 (7.9%)	8 (5.6%)	140 (7.7%)
Scapula	30 (1.8%)	0	30 (1.6%)
Humerus	230 (13.7%)	11 (7.7%)	241 (13.2%)
Forearm	394 (23.5%)	53 (37.1%)	447 (24.6%)
Hand	305 (18.2%)	35 (24.5%)	340 (18.7%)
Spine	86 (5.1%)	0	86 (4.7%)
Pelvis	48 (2.9%)	0	48 (2.6%)
Femur	153 (9.1%)	3 (2.1%)	156 (8.6%)
Patella	4 (0.2%)	1 (0.7%)	5 (0.3%)
Tibia	90 (5.4%)	29 (20.3%)	119 (6.5%)
Ankle	192 (11.4%)	2 (1.4%)	194 (10.7%)
Foot	13 (0.8%)	1 (0.7%)	14 (0.8%)

Number of fractures per anatomical region and their proportion of all sustained fractures for respective group

### Fracture distribution with regard to sex

In adults, there were 1082 fractures in women and 595 fractures in men. Wrist fractures were most common for women while hand fractures were the most frequent in men. Comparing the fracture distribution between the two groups, the biggest difference found were for wrist fractures that accounted for 27.8% of fractures in women vs. 8.1% in men. Proximal femoral fractures accounted for 4.6% of fractures in adult women and 14.8% of fractures in adult men. There were 16 adult fractures of the distal humerus registered, all occurring in women.

In children and adolescents, there was an equal distribution of fractures between boys and girls (*n* = 69, 48.3% vs. *n* = 74, 51.7%). Fractures of the wrist were the most common injury in both groups (*n* = 25, 36.2% for girls and *n* = 25, 33.8% for boys).

### Fracture distribution with regards to age

Most of the fractures (*n* = 1677, 92%) were among adults, and the median age for fractures in adults was 57 years (IQR 20). The median age for fractures in adult males was 56 years (IQR 30) and in females 58 years (IQR 20).

For children and adolescents, the median age was 11 years (IQR 4), with 12 years (IQR 6) for boys and 11 years (IQR 5) for girls.

**Fig. 2:**
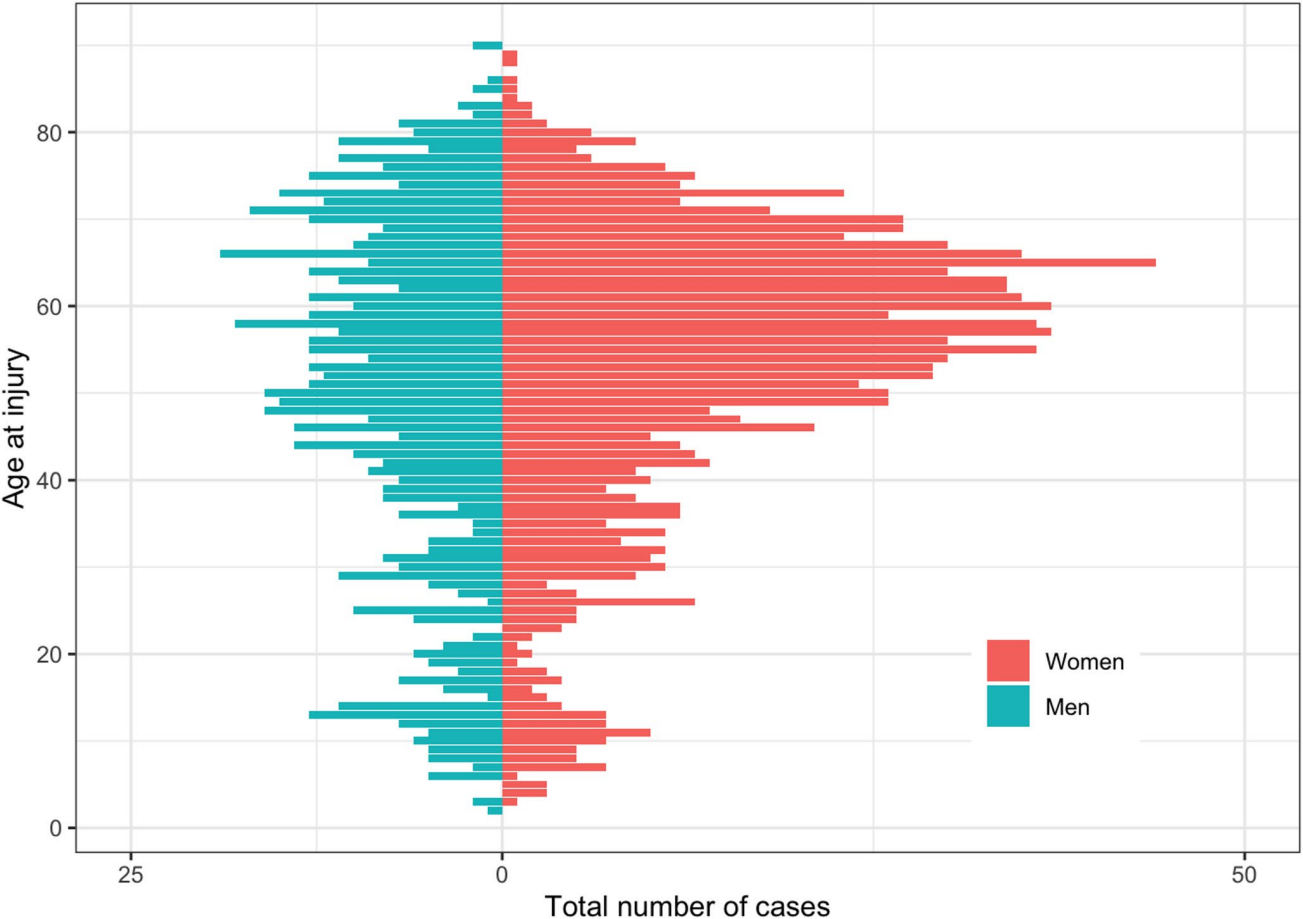
Age and sex distribution in 1820 fractures sustained while cross-country skiing: Age is on Y-axis and total number of cases on X-axis

There was no major difference between men and women with respect to number of fractures up to around 50 years of age. After 50 years of age, there were more registered fractures in women (Fig. 2). Among adults, injuries in women were more common with 1082 (64.5%) fractures versus 595 in men (35.5%).

### Surgically treated fractures

Some 560 fractures (31%) were treated operatively. The most common fracture subject to operative treatment overall was of the forearm, including the distal radius, with 171 fractures. The highest proportion of operatively treated fractures was found in femoral fractures, where surgery was performed in 144 injuries (96% of femoral fractures with treatment data registered). Pelvic fractures, all of which were fractures to the pubic rami, were all treated non-operatively (Table 3).

**Table 3:** Distribution of fractures treated surgically following cross-country skiing accidents

Anatomical Region	Adults (*n* = 536)	Children (*n* = 24)	Overall (*n* = 560)
Clavicle	19 (15.1%)	1 (12.5%)	20 (14.9%)
Scapula	4 (15.4%)	0	4 (15.4%)
Humerus	44 (20.4%)	2 (20.0%)	46 (20.4%)
Forearm	166 (42.8%)	5 (9.6%)	171 (38.9%)
Hand	36 (12.5%)	4 (12.5%)	40 (12.5%)
Spine	4 (4.9%)	0	4 (4.9%)
Pelvis			
Pelvic Ring	0	0	0
Acetabulum	4 (57.1%)	0	4 (57.1%)
Femur	142 (96.6%)	2 (66.7%)	144 (96.0%)
Patella	2 (50.0%)	0	2 (40.0%)
Tibia	37 (43.0%)	9 (31.0%)	46 (40.0%)
Ankle	77 (41.4%)	1 (100%)	78 (41.7%)
Foot	1 (7.7%)	0	1 (7.1%)

The proportion in percent describes how many of the fractures that were treated surgically per the specific anatomic region. In 77 out of 1820 fractures (4.2%) no treatment data was registered and these fractures were hence excluded in the analysi

### Seasonal variation of injuries

**Fig. 3:**
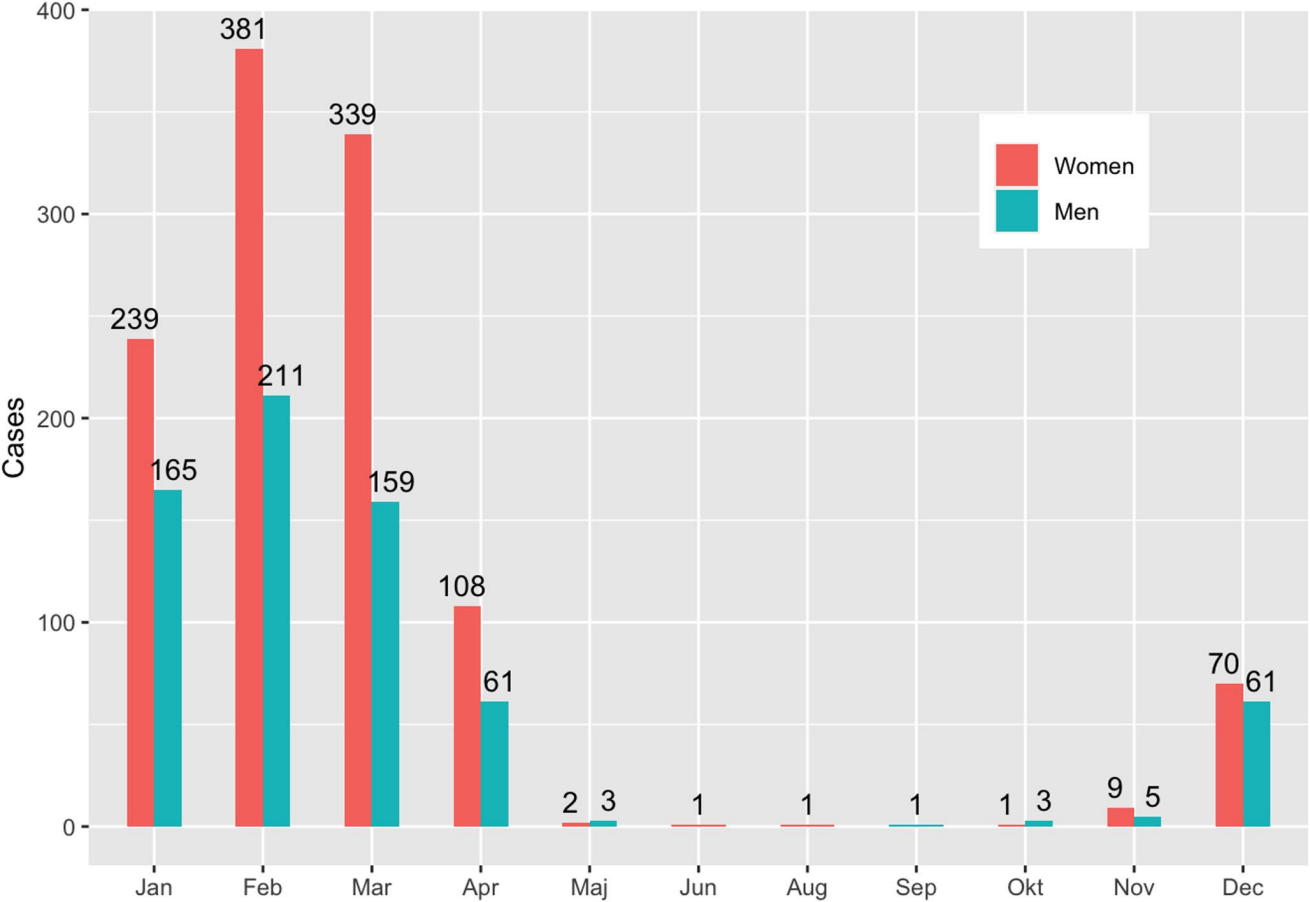
Number (n) of fractures sustained by cross-county skiing for women and men per month of the year

Unsurprisingly, the vast majority of the fractures occurred during winter months (December through April). February was the month with the most injuries reported with 592 (32.5%) fractures (Fig. 3).

## Discussion

During the study period, including 8 winter seasons, CCS accidents resulted in 1820 registered fractures. Most of these occurred in the upper extremity and injuries to the forearm and hand accounted for 43% of all fractures. Females sustained 63% of fractures and adult women sustained more fractures than adult men. In children and adolescents, both sexes were similarly affected. The most common injury for adult women, children and adolescents were wrist fractures. For adult men, hand fractures were most common. The surgical burden was relatively low with a total of 560 fractures, less than one-third of the total number of fractures, subject to operative treatment.

Fractures from simple same-level falls on ice and snow show a similar distribution as ours, with 60% upper extremity fractures and 69% of injuries occurring in females [12] .

Previous studies have reported that the most frequent injuries sustained when practicing CCS are sprains and bruises [1, 2, 8, 21]. In a study examining self-reported injuries in elite cross-country skiers, injuries to the lower extremities were more common [22] contradictory to our findings where the upper extremity was most commonly injured. This study examined injuries rather than fractures specifically and it is also reasonable to believe that the injury panorama observed in elite athletes differ from that found in the general population. Recreational skiers far more often suffer from injuries when practicing CCS [10], making comparisons difficult. There are few reports overall on the fracture topic and the existing studies are often dated.

As expected, injuries were mainly reported during the snow-rich winter months. Even though February is the shortest month of the year it has the most reported injuries. A possible explanation for this is the winter holiday in Swedish schools that occur during weeks 7–10 of the year.

As CCS requires snow, there should be a very limited potential to perform the sport outside of the winter months. There is however a variant where a pair of skis are connected to wheels (so-called ‘roller skis’) to mimic the sport. However, since the terrain differs, the injuries may differ substantially. Consideration should be taken if some cases on roller skis have been registered to SFR as injuries sustained while CCS.

Two thirds of all fractures occurred in female skiers. Most fractures among women were seen between the ages of 50 to 70 years. There was a significant increase in the number of fractures in this age group compared to younger age groups. In younger skiers, the fractures were rather evenly distributed between the sexes. For men, the fractures were more evenly spread throughout the ages. Speculating on potential underlying reasons as to why women sustain more fractures during CCS, one possible factor is the higher prevalence of osteoporosis among the female population, and particularly so in the Nordic countries. Even though osteoporosis among women is more prevalent post menopause, women also have a significantly higher lifetime risk of fractures than men at the age of 50. Osteoporosis is often associated with fractures to the wrist [23]. This speaks for the potential relevance of osteoporosis as a factor for sustaining a fracture while cross-country skiing. This hypothesis could be further investigated in future studies.

The most common fracture for children and adolescents were wrist fractures. For other sports such as snowboard, the use of wrist protectors have been proven to prevent this particular fracture [24]. The use of such protective equipment is not in place in CCS, but its introduction could be discussed given the results of our study as a way of preventing injuries. However, it could hinder the use of poles. Future studies could examine whether the use of such protective equipment could reduce the number of wrist fractures or alter the distribution of fractures.

A ski binding combining a mechanical component with an electrical component to reduce injuries has been developed in alpine skiing [25]. The use of such technology in CCS is not yet as far developed, but the use of new equipment has previously played an important part in the reduction of injuries and is likely to do so in the future. Other factors that could potentially help reduce the number of injuries are introductory courses for beginner skiers, proper preparation and maintenance of equipment and access to well prepared and lit ski-tracks designed with injury prevention in mind.

### Strengths and weaknesses

The major strength of this study is the large study population, allowing for an adequately large sample size to describe the distribution of fractures following CCS injuries. Furthermore, the SFR is a reliable source of demographic data, fracture classification and choice of treatment with increasing coverage during the study period and a substantial completeness of registrations. The SFR started in 2011 to register injury mechanism, fracture classification and treatment to improve fracture care. Outcome is evaluated by collecting data on reoperations and PROMs. This design collects basic variables for fracture outcome assessment but does not capture data on variables such as exposure time, skill-level, alcohol intake etc., limiting the interpretability of our finding.

As there are two main techniques in CCS, the skating technique, and the classical technique, it would have been preferred to distinguish between the two and make a comparison possible. The biomechanics of the two techniques differ markedly, and it is reasonable to suspect that this might translate into different injury panoramas depending on technique used. Unfortunately, there is currently no way to classify the technique used in the variables collected in SFR. Similarly, as exposure time is not registered, we are unable to determine the incidence of injuries per exposure hour.

The SFR only registers fractures to the spine and extremities. Fractures to the thorax and skull are not registered, nor are injuries to the abdomen, thorax and head. Non-osseous injuries to the spine and extremities like sprains and ligament injuries are also left out. Hence, the true burden of injuries following CCS accidents is not described in the present study. Furthermore, for children the recommendation is to only register fractures to the long bones, i.e. upper and lower arm, femur and tibia, since there are specific paediatric fracture classifications for the other locations. Therefore, comparisons between children and adults regarding fracture distribution should be interpreted cautiously.

As with all registers, the SFR is dependent on its users and their correct registration. Misregistration, incompleteness and misclassifications are always potential error sources when data from registers are used. Roller skis could make out some of the registrations in injuries occurring during the summer months, but they are few and should not affect the distribution of fractures in this large cohort. Also, some areas of Sweden have potential for very long CCS seasons. Given the large number of fractures registered, we believe the date provides an accurate overview of the injury panorama of fractures sustained in cross-country skiing accidents.

## Conclusion

Fractures sustained from accidents while cross-country skiing in Sweden most commonly affect the upper extremity. Fractures occur more frequently in females than in men. Most fractures are treated non-operatively.

The age and sex distribution suggests a possible link to osteoporosis. These findings identify women > 50 as a risk group. Recommendations regarding equipment, preventive precautions and training well in advance of intensive winter weeks should be distributed or communicated via relevant public health entities to try and mitigate the impact of these injuries.

## Data Availability

Due to legislation with respect to register data and restrictions stipulated in the ethical permission, the dataset analysed in this study is not freely available. Data can be obtained from the Center of Registers, Västra Götaland, Sweden if ethical approval is granted.

## References

[1] Langer PR (2017) Cross-Country Skiing. In: Werd MB, Knight EL, Langer PR (eds) Athletic footwear and orthoses in sports medicine. Springer International Publishing, Cham, pp 367–380 10.1007/978-3-319-52136-7_27

[2] Renstrom P, Johnson RJ (1989) Cross-country skiing injuries and biomechanics. Sports Med Auckl NZ 8(6):346–370 10.2165/00007256-198908060-00004 2694282

[3] Smith M, Matheson GO, Meeuwisse WH (1996) Injuries in cross-country skiing: a critical appraisal of the literature. Sports Med Auckl NZ 21(3):239–250 10.2165/00007256-199621030-00006 8776011

[4] Farahmand BY, Ahlbom A, Ekblom O, Ekblom B, Hållmarker U, Aronson D et al (2003) Mortality amongst participants in vasaloppet: a classical long-distance ski race in Sweden. J Intern Med 253(3):276–283 12603494 10.1046/j.1365-2796.2003.01122.x

[5] Laukkanen JA, Kunutsor SK, Ozemek C, Mäkikallio T, Lee DC, Wisloff U et al (2019) Cross-country skiing and running’s association with cardiovascular events and all-cause mortality: a review of the evidence. Prog Cardiovasc Dis 62(6):505–514 31505192 10.1016/j.pcad.2019.09.001

[6] Laukkanen JA, Laukkanen T, Kunutsor SK (2018) Cross-country skiing is associated with lower all-cause mortality: a population-based follow-up study. Scand J Med Sci Sports 28(3):1064–1072 28921697 10.1111/sms.12980

[7] Laukkanen JA, Lakka TA, Ogunjesa BA, Kurl S, Kunutsor SK (2020) Cross-country skiing and the risk of acute myocardial infarction: a prospective cohort study. Eur J Prev Cardiol 27(10):1108–1111 31422677 10.1177/2047487319869696

[8] Ristolainen L, Heinonen A, Turunen H, Mannström H, Waller B, Kettunen JA et al (2010) Type of sport is related to injury profile: a study on cross country skiers, swimmers, long-distance runners and soccer players. A retrospective 12-month study. Scand J Med Sci Sports 20(3):384–393 19602191 10.1111/j.1600-0838.2009.00955.x

[9] Nagle KB (2015) Cross-Country skiing injuries and training methods. Curr Sports Med Rep 14(6):442–447 26561764 10.1249/JSR.0000000000000205

[10] Ketterl R (2014) Breiten- und vereinssport beim Nordischen skisport. Unfallchirurg 117(1):33–40 24390721 10.1007/s00113-013-2466-2

[11] Han Pda, Gao D, Liu J, Lou J, Tian S, jia, Lian H, xin et al (2022) Medical services for sports injuries and illnesses in the Beijing 2022 olympic winter games. World J Emerg Med 13(6):459–466 36636567 10.5847/wjem.j.1920-8642.2022.106 PMC9807383

[12] Ivdal H, Bergenholtz L, Bergdahl C, Wolf O, Rydberg EM (2025) Fractures sustained by slipping on ice or snow: an epidemiological study of 50,500 fractures from the Swedish fracture register. Acta Orthop 96:272–277 40134286 10.2340/17453674.2025.43186 PMC11933825

[13] Möller M, Wolf O, Bergdahl C, Mukka S, Rydberg EM, Hailer NP et al (2022) The Swedish fracture register - ten years of experience and 600,000 fractures collected in a National quality register. BMC Musculoskelet Disord 23(1):141 35148730 10.1186/s12891-022-05062-w PMC8832767

[14] Marsh JL, Slongo TF, Agel J, Broderick JS, Creevey W, DeCoster TA et al (2007) Fracture and dislocation classification compendium – 2007: orthopaedic trauma association classification, database and outcomes committee. J Orthop Trauma 21(10 Suppl):S1–133 18277234 10.1097/00005131-200711101-00001

[15] Knutsson SB, Wennergren D, Bojan A, Ekelund J, Möller M (2019) Femoral fracture classification in the Swedish fracture Register – a validity study. BMC Musculoskelet Disord 20(1):197 31068172 10.1186/s12891-019-2579-z PMC6506935

[16] Bergvall M, Bergdahl C, Ekholm C, Wennergren D (2021) Validity of classification of distal radial fractures in the Swedish fracture register. BMC Musculoskelet Disord 22(1):587 10.1186/s12891-021-04473-5 PMC8235642 34174861

[17] Juto H, Möller M, Wennergren D, Edin K, Apelqvist I, Morberg P (2016) Substantial accuracy of fracture classification in the Swedish fracture register: evaluation of AO/OTA-classification in 152 ankle fractures. Injury 47(11):2579–2583 27645617 10.1016/j.injury.2016.05.028

[18] Bergdahl C, Nilsson F, Wennergren D, Ekholm C, Möller M (2021) Completeness in the Swedish fracture register and the Swedish National patient register: an assessment of humeral fracture registrations. Clin Epidemiol 13:325–333 34045902 10.2147/CLEP.S307762 PMC8149280

[19] von Elm E, Altman DG, Egger M, Pocock SJ, Gøtzsche PC, Vandenbroucke JP et al (2014) The strengthening the reporting of observational studies in epidemiology (STROBE) statement: guidelines for reporting observational studies. Int J Surg Lond Engl 12(12):1495–1499

[20] R Core Team. R: a language and environment for statistical computing. R Foundation for Statistical Computing, Vienna, Austria. (2023) Available from: https://www.R-project.org/

[21] Butcher JD, Brannen SJ (1998) Comparison of injuries in classic and skating nordic ski techniques. Clin J Sport Med 8(2):88–91 9641435 10.1097/00042752-199804000-00004

[22] Worth SGA, Reid DA, Howard AB, Henry SM (2019) Injury incidence in competitive cross-country skiers: a prospective cohort study. Int J Sports Phys Ther 14(2):237–252 30997276 PMC6452571

[23] Keen MU, Reddivari AKR (2023) Osteoporosis in Females. In: StatPearls. StatPearls Publishing, Treasure Island (FL). Available from: http://www.ncbi.nlm.nih.gov/books/NBK559156/

[24] Rønning R, Rønning I, Gerner T, Engebretsen L (2001) The efficacy of wrist protectors in preventing snowboarding injuries. Am J Sports Med 29(5):581–585 11573916 10.1177/03635465010290051001

[25] Hermann A, Senner V (2021) Knee injury prevention in alpine skiing. A technological paradigm shift towards a mechatronic ski binding. J Sci Med Sport 24(10):1038–1043 32631774 10.1016/j.jsams.2020.06.009

